# Supplementary material for: Constraints on the optimization of gene product diversity
Source: Mol Syst Biol. 2025 Apr 10;21(5):472–91. doi: 10.1038/s44320-025-00095-4 (PMC12048591; doi:10.1038/s44320-025-00095-4)
Supplement: Supplementary file 2 — Expanded View Figures [file 44320_2025_95_MOESM2_ESM.pdf]

Expanded View Figures

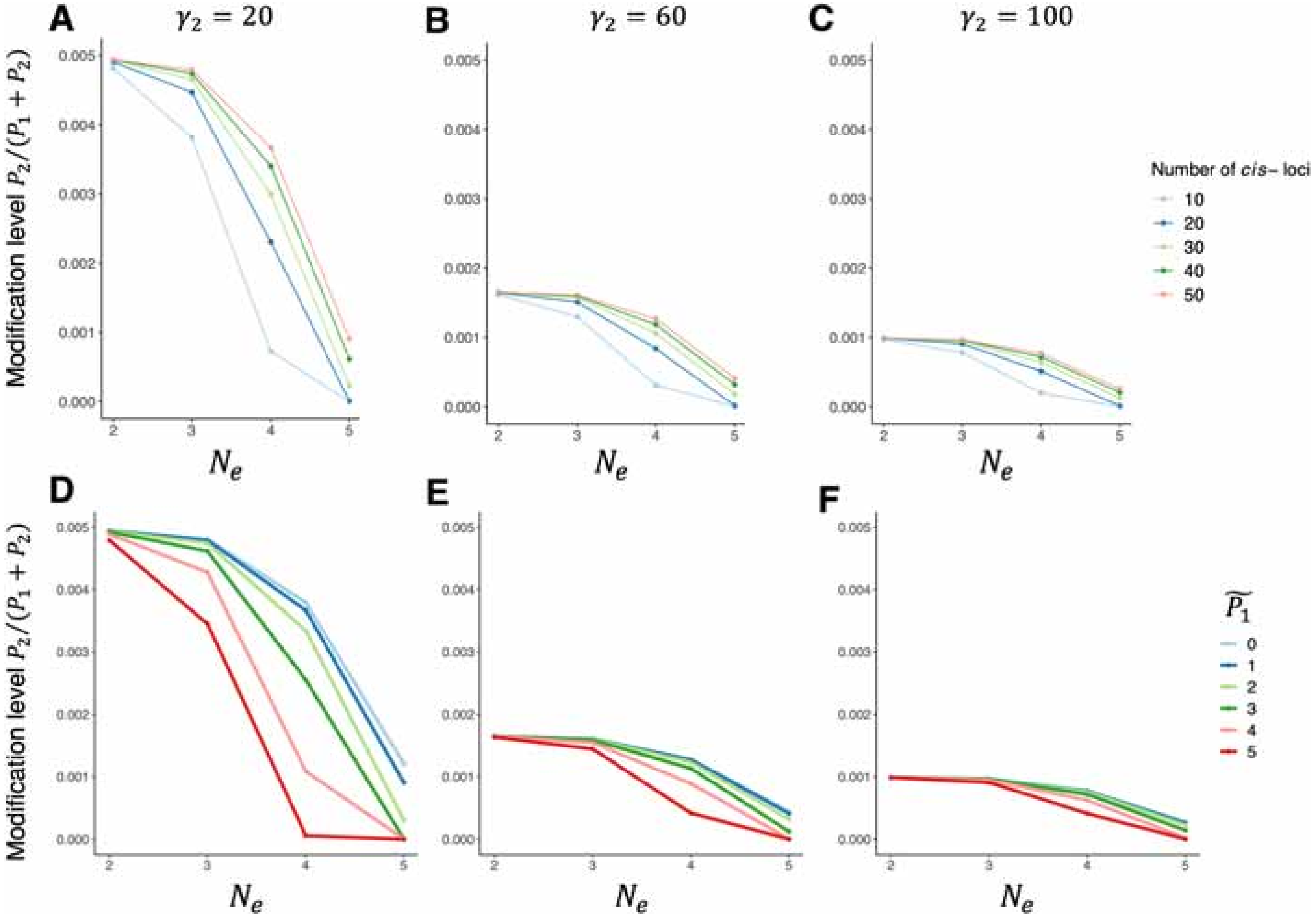

Figure EV1. Mean modification level varies with population genetic environment and genetic architecture.

Scaling between mean modification level of splicing-type modification and effective population size N_e (shown in log10 scale). (**A–C**) Response of mean modification level to N_e under different combinations of *cis*-loci number (l) and decay rates of the dysfunctional isoform (γ_2), with optimal expression level $\tilde{P}_1 = \exp(1)$ ($\ln \tilde{P}_1 = 1$). (**D–F**) Response of mean modification level to N_e under different $\tilde{P}_1 = \frac{\alpha}{\gamma_1}$ and γ_2, with $l = 50$. All results are derived with initial *cis*-genotypic value $v_O = l$, with $T = 10^8$ time steps, $\mu_{01} = \mu_{10} = 10^{-8}$, $Q = 100$, $\gamma_O = 0$, $\gamma_1 = 1$, and $\tilde{P}_1 = \alpha/\gamma_1$.

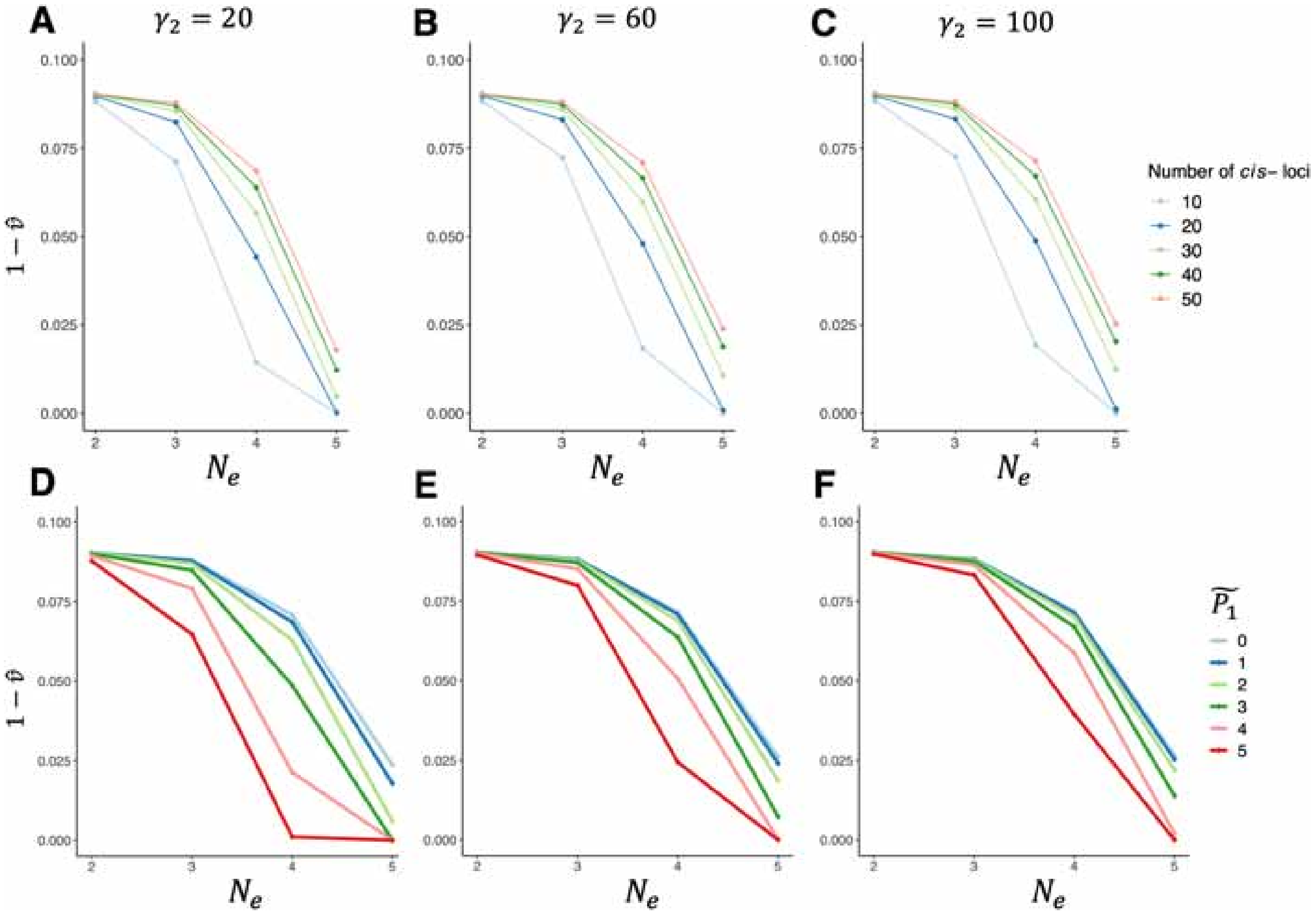

Figure EV2. *Cis*-genotypic value varies with population genetic environment and genetic architecture.

Scaling between normalized mean *cis*-genotypic value of splicing-type modification and N_e (shown in log10 scale). Represented by the Y-axes is $1 - \hat{v}$, which reflects the degree to which *cis*-genotype favors production of the dysfunction and toxic isoform I_2. **(A–C)** Response of $1 - \hat{v}$ to N_e under different combinations of l and γ_2, with optimal expression level $\tilde{P}_1 = \exp(1)$ ($\ln \tilde{P}_1 = 1$). **(D–F)** Response of $1 - \hat{v}$ to N_e under different $\tilde{P}_1$ and γ_2, with $l = 50$. All results are derived with initial *cis*-genotypic value $v_0 = l$, time of evolution $T = 10^8$ time steps, and $\mu_{01} = \mu_{10} = 10^{-8}$, $Q = 100$, $\gamma_0 = 0$, $\gamma_1 = 1$, and $\tilde{P}_1 = \alpha/\gamma_1$.

 © The Author(s)

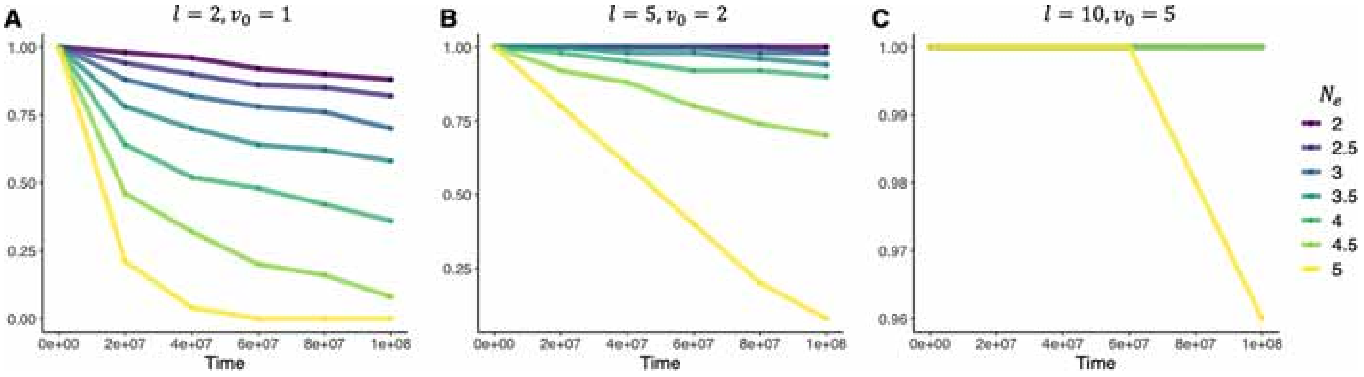

Figure EV3. Conservation of modification events as a function of time since divergence.

(**A**) $l = 2$, $v_O = 1$. (**B**) $l = 5$, $v_O = 2$. (**C**) $l = 10$, $v_O = 5$. Y-axes represent among-gene median of proportion of lineages (species) that share a modification event when selection on Q is weak ($\sigma_Q = 20$). When two curves in the same panel completely overlap, the one with the largest corresponding N_e is shown.

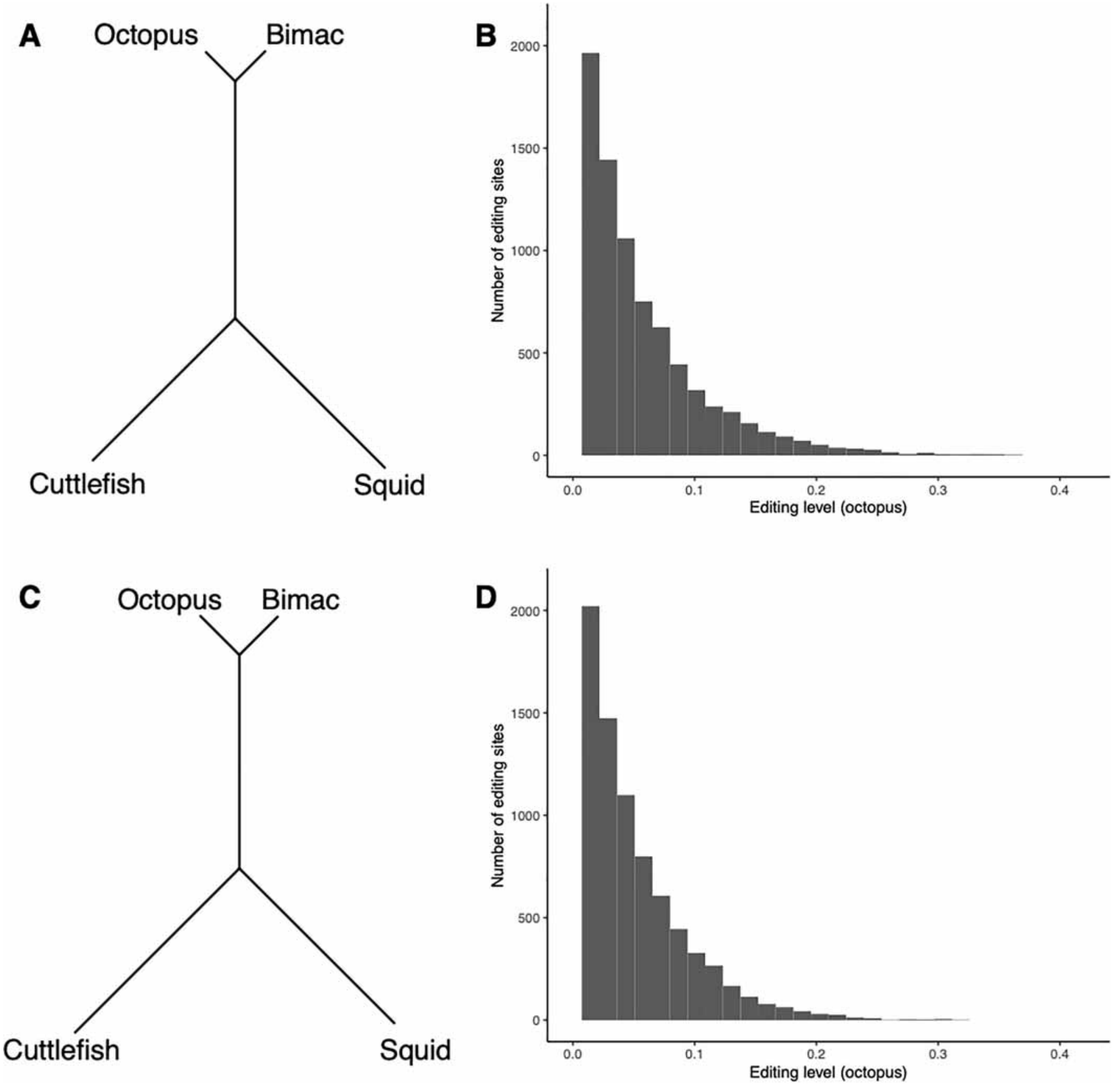

Figure EV4. Simulations of A-to-I RNA editing along the coleoid phylogeny.

(**A**) Neighbor-joining tree of four coleoid species based on simulated neutral editing levels. (**B**) Distribution of neutral editing levels in the octopus. (**C**) Neighbor-joining tree of four coleoid species based on simulated deleterious editing levels. (**D**) Distribution of deleterious editing levels in the octopus.

 © The Author(s)
